# Association of Serum Aluminum Levels with Mortality in Patients on Chronic Hemodialysis

**DOI:** 10.1038/s41598-018-34799-5

**Published:** 2018-11-13

**Authors:** Ming-Hsien Tsai, Yu-Wei Fang, Hung-Hsiang Liou, Jyh-Gang Leu, Bing-Shi Lin

**Affiliations:** 1 Division of Nephrology, Department of Internal Medicine, Shin Kong Wu Ho-Su Memorial Hospital, Taipei, ROC Taiwan; 2 Fu-Jen Catholic University School of Medicine, Taipei, ROC Taiwan; 3 Division of Biostatistics, Institutes of Epidemiology and Preventive Medicine, College of Public Health, National Taiwan University, Taipei, ROC Taiwan; 4 Division of Nephrology, Department of Internal Medicine, Hsin-Jen Hospital, New Taipei City, ROC Taiwan

## Abstract

Despite reported evidence on the relationship between higher serum aluminum levels and poor outcomes in patients on chronic hemodialysis (CHD), the acceptable cutoff value of serum aluminum for mortality remains unclear. A retrospective observational cohort study with 636 Taiwanese patients on CHD was conducted to investigate the impact of serum aluminum levels on mortality. The predictors were bivariate serum aluminum level (<6 and ≥6 ng/mL) and the Outcomes were all-cause and cardiovascular (CV) mortality. During the mean follow-up of 5.3 ± 2.9 years, 253 all-cause and 173 CV deaths occurred. Crude analysis showed that a serum aluminum level of ≥6 ng/mL was a significant predictor of all-cause [hazard ratio (HR), 1.80; 95% confidence interval (CI), 1.40–2.23] and CV (HR, 1.84; 95% CI, 1.36–2.50) mortality. After multivariable adjustment, the serum aluminum level of ≥6 ng/mL remained a significant predictor of all-cause mortality (HR, 1.37, 95% CI, 1.05–1.81) but became insignificant for CV mortality (HR, 1.29; 95% CI, 0.92–1.81). Therefore, our study revealed that a serum aluminum level of ≥6 ng/mL was independently associated with all-cause death in patients on CHD, suggesting that early intervention for aluminum level in patients on CHD might be beneficial even in the absence of overt aluminum toxicity.

## Introduction

Identifying risk factors for mortality may help in early intervention approaches to improve the survival of patients on chronic hemodialysis (CHD) who have a substantially reduced life expectancy^1,2^. Controlling aluminum levels is an important issue for patients with chronic kidney disease (CKD) because systemic aluminum toxicity is harmful^3^. Moreover, an elevated serum aluminum level can lead to dialysis dementia^4^, osteomalacia, a very low bone turnover rate with marked accumulation of unmineralized osteoid^5^, iron-resistant microcytic anemia^6^ and cardiomegaly^7^ in dialysis patients.

Currently, severe aluminum toxicity (serum aluminum level >200 ng/mL) in patients on CHD is uncommon^8,9^ due to the removal of aluminum from water used for dialysis by reverse osmosis and deionization as well as the use of widely available nonaluminum-containing phosphate binders. However, controlling serum aluminum levels remains an important issue for patients on CHD. Aluminum removal by dialysis is not efficient, and the possible source of aluminum accumulation in patients on CHD is oral (aluminum-containing phosphate binders and antacids) and injectable medications (calcitriol, vitamins B complex, iron and erythropoietin) that are commonly administered to dialysis patients^10,11^. Therefore, the National Kidney Foundation–Kidney Disease Outcomes Quality Initiative (KDOQI) guidelines^12^ recommend that the baseline serum aluminum level should be below 20 ng/mL and that aluminum levels and risk for aluminum toxicity should be assessed at least once per year.

Chazan *et al*. demonstrated that elevated aluminum levels are associated with mortality in a study on 10646 patients on maintenance CHD. The annual mortality rate was 18% higher for patients with serum aluminum levels between 40.9 and 59.8 ng/mL and progressively increased to 60% higher for those with aluminum levels above 199.7 ng/mL than for those with levels below 38.9 ng/mL^13^. One recent study reported that patients on CHD with serum aluminum levels more than 9 ng/mL had significantly poorer outcomes than those with levels below 6 ng/mL over a year of observation^14^, demonstrating that aluminum, even within an apparently acceptable range (i.e., <20 ng/mL), is also associated with increased mortality in patients on CHD. However, a significant difference between the groups with serum aluminum levels below 6 ng/mL and 6–9 ng/mL was not observed in that study^14^. Moreover, the evidence on the serum aluminum cutoff value to assess its clinical significance in association with mortality in patients on CHD remains unclear due to the insufficient observation time and insufficient adjustment for risk factors.

Therefore, we conducted a retrospective observational cohort study to test the effect of a serum aluminum cutoff value of 6 ng/mL (upper normal limit) on all-cause and cardiovascular mortality in patients on CHD.

## Methods

### Study design and patients

This retrospective observational cohort study was conducted at a single medical center, Shin Kong Wu Ho-Su Memorial Hospital, and used the medical records of patients undergoing hemodialysis from December 2006 to December 2012. Medical records of only those patients who were receiving regular hemodialysis for at least 3 months before data collection and those who were clinically stable without hospitalization during the 3 months preceding data collection were included. Initially, 805 patients were eligible for this study. Furthermore, the medical records of patients with unavailable serum aluminum levels were excluded. Finally, the records of 636 patients on CHD were selected. Patient outcomes were observed until December 2015. Patients who died at the hospital during follow-up were identified from the discharge diagnosis and death certificates in hospital charts, which the causes of death were classified into CV event, cerebrovascular event, gastrointestinal bleeding event, and unknown etiology by the attending physician of nephrologist. Patients who were transferred to other dialysis centers, switched to peritoneal dialysis, or received renal transplantation were censored.

This study was performed in accordance with the principles of the Declaration of Helsinki and was approved by the Ethics Committee of the Shin Kong Wu Ho-Su Memorial Hospital. Informed consent was waived because the study was based on a medical chart review. Patient information was protected by anonymization and de-identification prior to analysis.

### Demographic and laboratory data

Demographic and laboratory data were obtained from the medical records and included age; sex; hemodialysis vintage; cardiothoracic ratio (CTR); levels of aluminum, blood urea nitrogen, serum creatinine, albumin, uric acid, total cholesterol, triglycerides, hemoglobin, intact parathyroid hormone, ionized calcium, serum phosphate, and alkaline phosphatase; iron profile; urea kinetics; history of diabetes mellitus (DM), hypertension, coronary artery disease, or cerebrovascular disease; and prescription of renin–angiotensin system blockers, lipid-lowering agents, beta-blockers, and antiplatelet agents. Coronary artery disease was defined as a history of exertional angina, significant arterial occlusive disease disclosed by an angiogram, past myocardial infarction, coronary artery bypass surgery, or angioplasty. Cerebrovascular disease was defined as a history of cerebrovascular accidents, either hemorrhage or infarction. CVD was diagnosed based on a documented history of coronary artery or cerebrovascular disease. Blood samples were collected before, following an at least 8-h fast, and immediately after the dialysis session. The blood samples post dialysis were used to assess the urea kinetics. Biochemical analyses were conducted using standard commercially available assays and automated test machines (Beckman Coulter, Lane Cove, NSW, Australia). Intact parathyroid hormone levels were measured using the Roche Elecsys assay (Roche Diagnostics, Basel, Switzerland). Aluminum levels were measured by graphite furnace atomic absorption spectrometry using GBC 906AA (Braeside VIC, Australia).

### Statistical analysis

Data are presented as mean ± standard deviation or median with interquartile range as appropriate for continuous data and number (%) for categorical data. Student’s *t-*test was used to compare the means of continuous variables, and the χ^2^ test was used for categorical variables. Continuous aluminum levels were natural-log transformed (ln) to approximate a normal distribution. Linear regression analyses were performed using ln(aluminum) as the dependent variable. Variables were chosen into the multivariable analysis by stepwise methods; *P-*values by *F*-statistic for entry and removal were <0.05 and >0.10, respectively. Moreover, serum aluminum levels were categorized into two groups based on the cutoff of 6 ng/mL. Survival curves were estimated using the Kaplan–Meier method and tested by the log-rank test. Cox proportional regression model was used to determine the risk of death. The assumption of proportionality was not violated by testing for the interaction between time and variables. Additionally, subgroup analyses were performed for the following variables: DM, age (≤60 and >60 years), sex, previous CVD, and ionized calcium level (≤4.5 and >4.5 mg/dL). A two-tailed *P-*value of <0.05 was considered statistically significant. All statistical analyses were performed using SAS for Windows version 9.4 (SAS Institute Inc., Cary, NC, USA).

## Results

**Figure 1:**
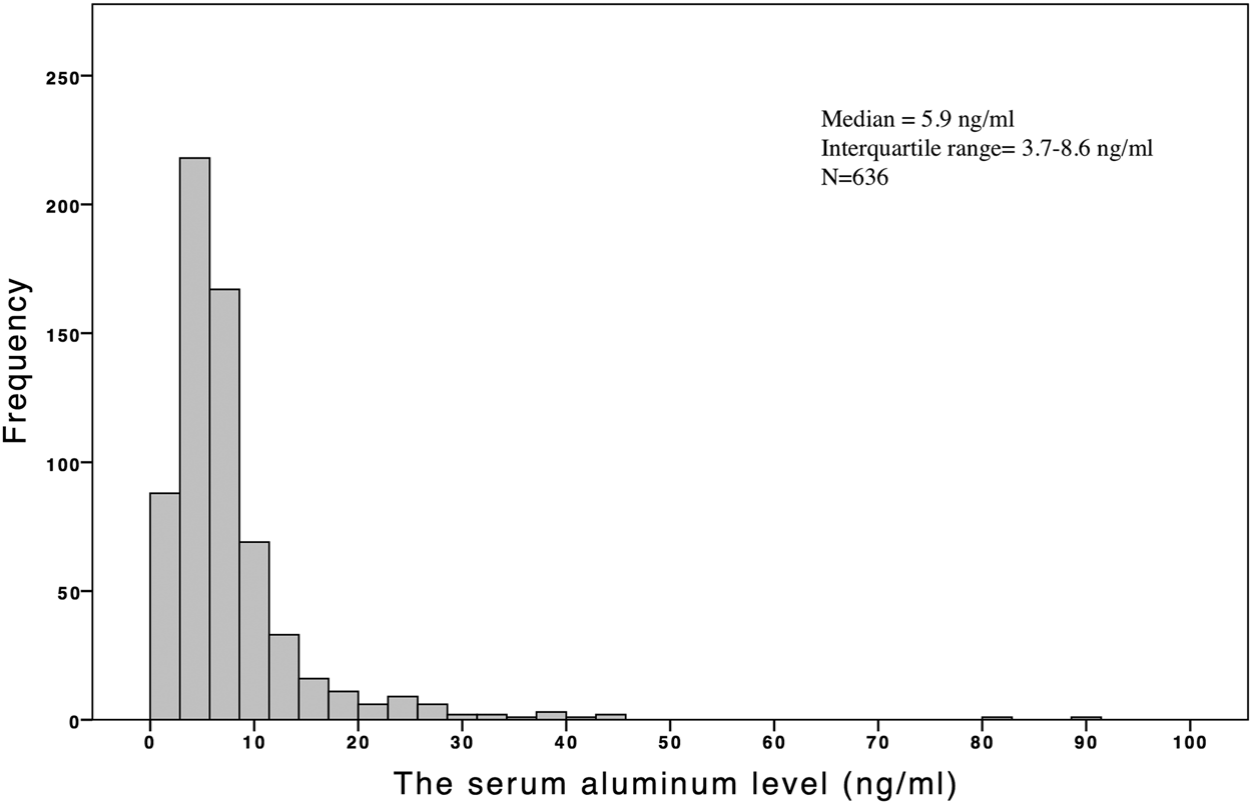
The distribution of serum aluminum levels in patients on chronic hemodialysis.

The mean age, length of follow-up, and mean HD vintage among the entire cohort of 636 patients on CHD were 62.8 ± 13.2 years, 5.3 ± 2.9 years, and 5.0 ± 4.7 years, respectively. Of the total, 47.8% were males and 38.5%, 39.9%, and 27.6% had DM, hypertension, and CVD, respectively (Table 1). Right-tail distribution of serum aluminum level was observed in the entire cohort (Fig. 1). Table 1 lists the demographic and clinical data of the participants stratified by the serum aluminum level (<6 and ≥6 ng/mL). Those with higher aluminum levels were older and predominantly females. Moreover, those with serum aluminum levels of ≥6 ng/mL had significantly higher alkaline phosphatase levels and CTR and lower creatinine, uric acid and albumin levels; the frequencies of antiplatelet and lipid-lowering agent prescriptions were also higher in this group.

**Table 1:** Baseline characteristics of the study population.

Characteristic	All (n = 636)	Al < 6 ng/mL (n = 322)	Al ≥ 6 ng/mL (n = 314)	*P*
Aluminum (ng/mL)	7.7 ± 7.6	3.7 ± 1.3	11.7 ± 9.1	NA
Age (years)	62.8 ± 13.2	61.6 ± 14.1	64.0 ± 12.1	0.021
Male sex	304 (47.8)	172 (53)	132 (42)	0.004
Duration of dialysis (years)	5.0 ± 4.7	4.6 ± 4.7	5.3 ± 4.8	0.078
Diabetes mellitus	244 (38.5)	114 (35)	130 (41)	0.143
Cardiovascular disease	175 (27.6)	87 (27)	88 (28)	0.832
Hypertension	254 (39.9)	121 (37)	133 (42)	0.218
Blood nitrogen (mg/dL)	69 ± 18	68 ± 17	69 ± 19	0.576
Creatinine (mg/dL)	9.5 ± 2.3	9.8 ± 2.2	9.3 ± 2.3	0.001
Uric acid (mg/dL)	6.6 ± 2.3	6.9 ± 1.9	6.3 ± 2.6	0.001
Albumin (g/dL)	4.1 ± 0.4	4.2 ± 0.4	4.1 ± 0.4	0.031
Triglyceride (mg/dL)	163 ± 149	155 ± 141	171 ± 157	0.180
Cholesterol (mg/dL)	175 ± 44	173 ± 40	177 ± 47	0.252
Kt/V	1.3 ± 0.2	1.3 ± 0.2	1.3 ± 0.2	0.429
Hemoglobin (g/dL)	10.4 ± 1.4	10.4 ± 1.4	10.3 ± 1.5	0.601
Transferrin saturation (%)	35.3 ± 15.2	36.2 ± 16.1	34.3 ± 14.2	0.116
Ionized calcium (mg/dL)	4.6 ± 0.4	4.6 ± 0.4	4.6 ± 0.4	0.123
Phosphate (mg/dL)	5.2 ± 1.4	5.1 ± 1.4	5.2 ± 1.4	0.397
Alkaline phosphatase (U/L)	98 ± 70	90 ± 57	107 ± 80	0.002
iPTH (pg/mL)	163 ± 199	158 ± 221	168 ± 174	0.510
Cardiothoracic ratio (%)	50.6 ± 6.7	49.3 ± 7.0	51.9 ± 6.2	<0.001
Medications				
Antiplatelet agents	220 (34.8)	99 (31)	121 (38)	0.044
RAS blockaders	219 (31.8)	119 (37)	100 (31)	0.149
Beta-blocker	116 (18.3)	56 (17)	60 (19)	0.613
Lipid-lowering agents	122 (19.2)	50 (15.6)	72 (22.9)	0.020

Values are expressed as number (%) of patients or as mean ± standard deviation Abbreviations: Al, aluminum; Kt/V, urea kinetics; iPTH, intact parathyroid hormone; NA, not available; RAS, renin–angiotensin system.

### Factors significantly associated with serum aluminum levels in patients on CHD

As summarized in Table 2, older age, female sex, longer HD vintage, higher ionized calcium and alkaline phosphatase levels, lower creatinine level, and higher CTR were significantly associated with higher ln(aluminum) levels in patients on CHD. Moreover, multiple linear regression analysis with a stepwise method for parameter selection demonstrated that ln(aluminum) levels exhibited a significantly positive association with HD duration, ionized calcium and alkaline phosphatase levels, and CTR.

**Table 2:** Determinants of serum log (aluminum level) by simple linear regression analysis.

Parameter	Crude		Multivariable	
	Estimate (95% CI)	*P*	Estimate (95% CI)	*P*
Age (per year)	0.005 (0.001, 0.009)	0.029		
Male versus female	−0.182 (−0.29, −0.072)	0.001		
Duration of dialysis (per year)	0.019 (0.008, 0.031)	0.001	0.012 (0.001, 0.025)	0.045
Diabetes mellitus	0.047 (−0.067, 0.160)	0.419		
Cardiovascular disease	0.088 (−0.035, 0.211)	0.160		
Hypertension	0.069 (−0.044, 0.181)	0.232		
Creatinine (mg/dL)	−0.030 (−0.054, 0.006)	0.015		
Uric acid (mg/dL)				
Hemoglobin (per g/dL)	0.006 (−0.032, 0.043)	0.769		
Albumin (per g/dL)	−0.118 (−0.256, 0.020)	0.094		
Kt/V (per unit)	0.082 (−0.161, 0.325)	0.508		
Ionized calcium (per mg/dL)	0.186 (0.067, 0.305)	0.002	0.135 (0.010, 0.261)	0.035
Phosphate (per mg/dL)	0.023 (−0.015, 0.061)	0.238		
ALK-P (per 10 U/L)	0.016 (0.008, 0.024)	<0.001	0.015 (0.006, 0.023)	0.001
iPTH (per 10 pg/mL)	0.002 (0.000, 0.005)	0.087		
Cardiothoracic ratio (per 1%)	0.021 (0.013, 0.029)	<0.001	0.019 (0.011, 0.027)	<0.001

Abbreviations: CI, confidence interval; Kt/V, urea kinetics; ALK-P, alkaline phosphatase; iPTH, intact parathyroid hormone.

### All-cause mortality in patients on CHD

**Figure 2:**
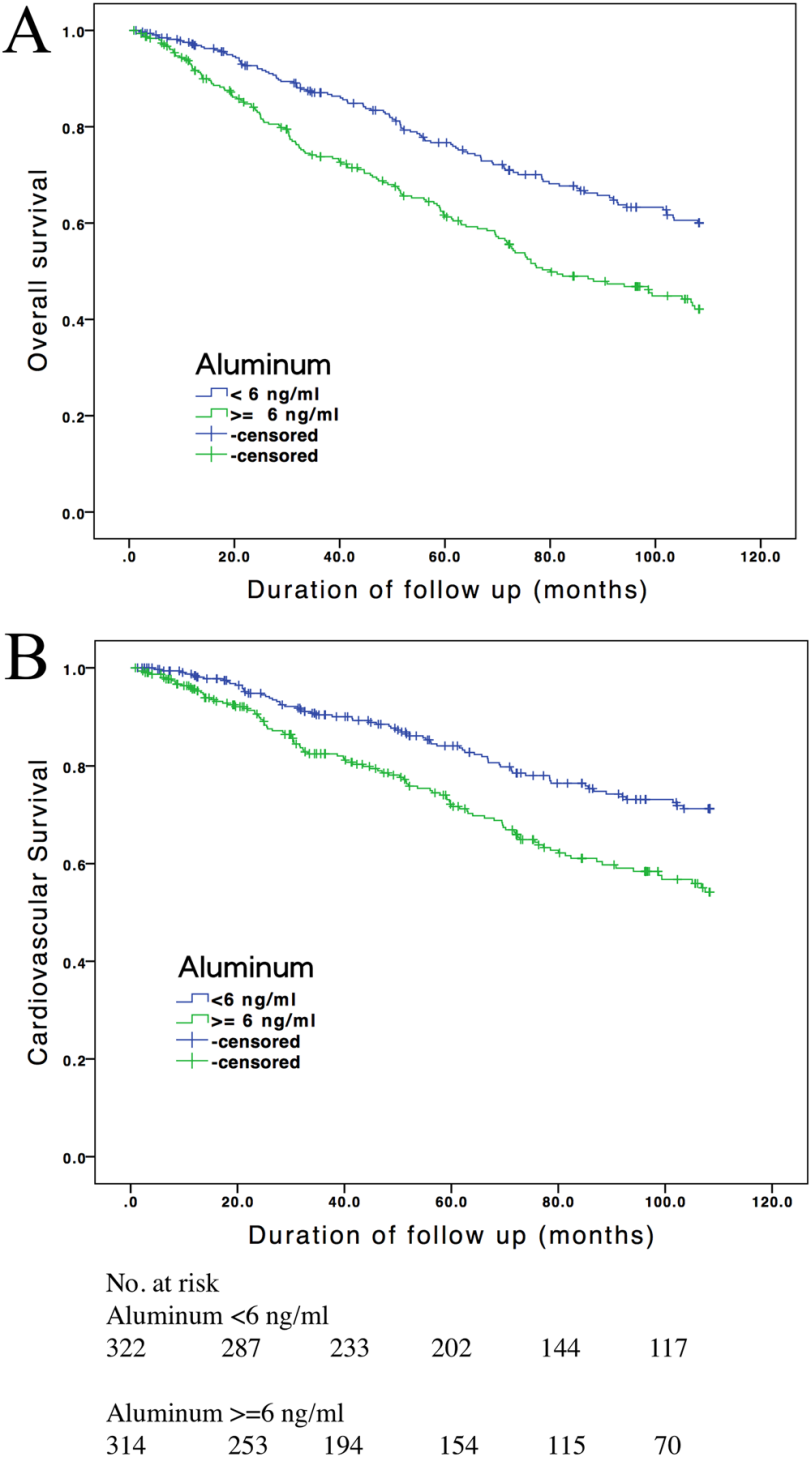
Probabilities of survival according to serum aluminum levels (<6 and ≥6 ng/mL) (**A**) in all-cause mortality with a log-rank test (χ^2^ = 20.4; *P* < 0.001) and (**B**) in cardiovascular mortality with a log-rank test (χ^2^ = 15.1; *P* < 0.001).

On follow-up, there were 253 (39.7%) deaths due to all causes, including 173 fatal CV events, 33 infectious diseases, 13 malignancies, 9 cerebrovascular events, 7 gastrointestinal bleeding events, and 18 deaths due to unknown etiology. Figure 2A shows the Kaplan–Meier survival curves of all-cause mortality according to the bivariate aluminum levels (<6 and ≥6 ng/mL). The difference in survival among the two groups was significant for all-cause mortality (χ^2^ = 20.4; *P* < 0.001). Results of the Cox proportional hazards regression analysis are shown in Table 2. The crude hazard ratio (HR) of the bivariate aluminum levels (<6 and ≥6 ng/mL) for all-cause mortality was 1.80 [95% confidence interval (CI), 1.40–2.32]. After multivariable adjustment, the bivariate aluminum levels (<6 and ≥6 ng/mL) remained a significant predictor of mortality (HR, 1.37; 95% CI, 1.05–1.81). Moreover, the continuous ln(aluminum) level was a significant predictor of all-cause mortality in both crude and multivariable analyses.

### Cardiovascular mortality in patients on CHD

There were 173 fatal CV events during the observation period of this study. Figure 2B shows the Kaplan–Meier survival curves of CV mortality according to the bivariate aluminum levels (<6 and ≥6 ng/mL). The difference in survival among the two groups was significant for all-cause mortality (χ^2^ = 15.1; *P* < 0.001). As presented in Table 3, the crude HR of the bivariate aluminum levels (<6 and ≥6 ng/mL) for CV mortality was 1.84 (95% CI, 1.36–2.50). After multivariable adjustment, HR (1.29; 95% CI, 0.92–1.81) became an insignificant predictor of CV mortality. The predictability for CV mortality of continuous ln (aluminum) level followed the same pattern as bivariate aluminum levels. However, the HRs of the bivariate aluminum levels (1.50; 95% CI, 1.07–2.09) and continuous ln (aluminum) (1.28; 95% CI, 1.03–1.60) for CV mortality became significant in the full multivariable model without adjusting the parameter of CTR.

**Table 3:** Multivariable Cox regression analysis of risk factor for mortality.

	Aluminum cutoff value of 6 ng/mL		Every 1 increment in ln(Aluminum)	
	Hazard ratio (95% CI)	*P* value	Hazard ratio (95% CI)	*P* value
All-cause mortality				
Crude	1.80 (1.40–2.32)	<0.001	1.48 (1.25–1.74)	<0.001
Mode 1	1.79 (1.39–2.31)	<0.001	1.45 (1.23–1.71)	<0.001
Mode 2	1.55 (1.19–2.02)	0.001	1.34 (1.12–1.59)	<0.001
Mode 3	1.37 (1.05–1.81)	0.023	1.25 (1.04–1.50)	0.015
Mode 4	1.56 (1.19–2.04)	0.001	1.37 (1.15–1.64)	<0.001
Cardiovascular mortality				
Crude	1.84 (1.36–2.50)	<0.001	1.47 (1.21–1.79)	<0.001
Mode 1	1.86 (1.36–2.53)	<0.001	1.46 (1.20–1.78)	<0.001
Mode 2	1.60 (1.16–2.20)	0.003	1.35 (1.10–1.67)	0.003
Mode 3	1.29 (0.92–1.81)	0.127	1.18 (0.94–1.48)	0.137
Mode 4	1.50 (1.07–2.09)	0.016	1.28 (1.03–1.60)	0.023

Multivariate model 1 is adjusted for age, sex, and hemodialysis vintage. Multivariate model 2 comprises model 1 as well as adjustments for diabetes mellitus, cardiovascular disease, and CTR. Multivariate model 3 comprises model 2 as well as adjustments for levels of creatinine, albumin, hemoglobin, ionized calcium, phosphate, alkaline phosphatase, and transferrin saturation; KT/V; as well as prescriptions of antiplatelet medications, renin–angiotensin system blockers, beta-blockers, and lipid-lowering agents. Multivariate model 4 comprises model 3 without CTR Abbreviations: CI, confidence interval; CTR, cardiothoracic ratio; Kt/V, urea kinetics; ln, natural log transformation.

### Subgroup analysis

**Figure 3:**
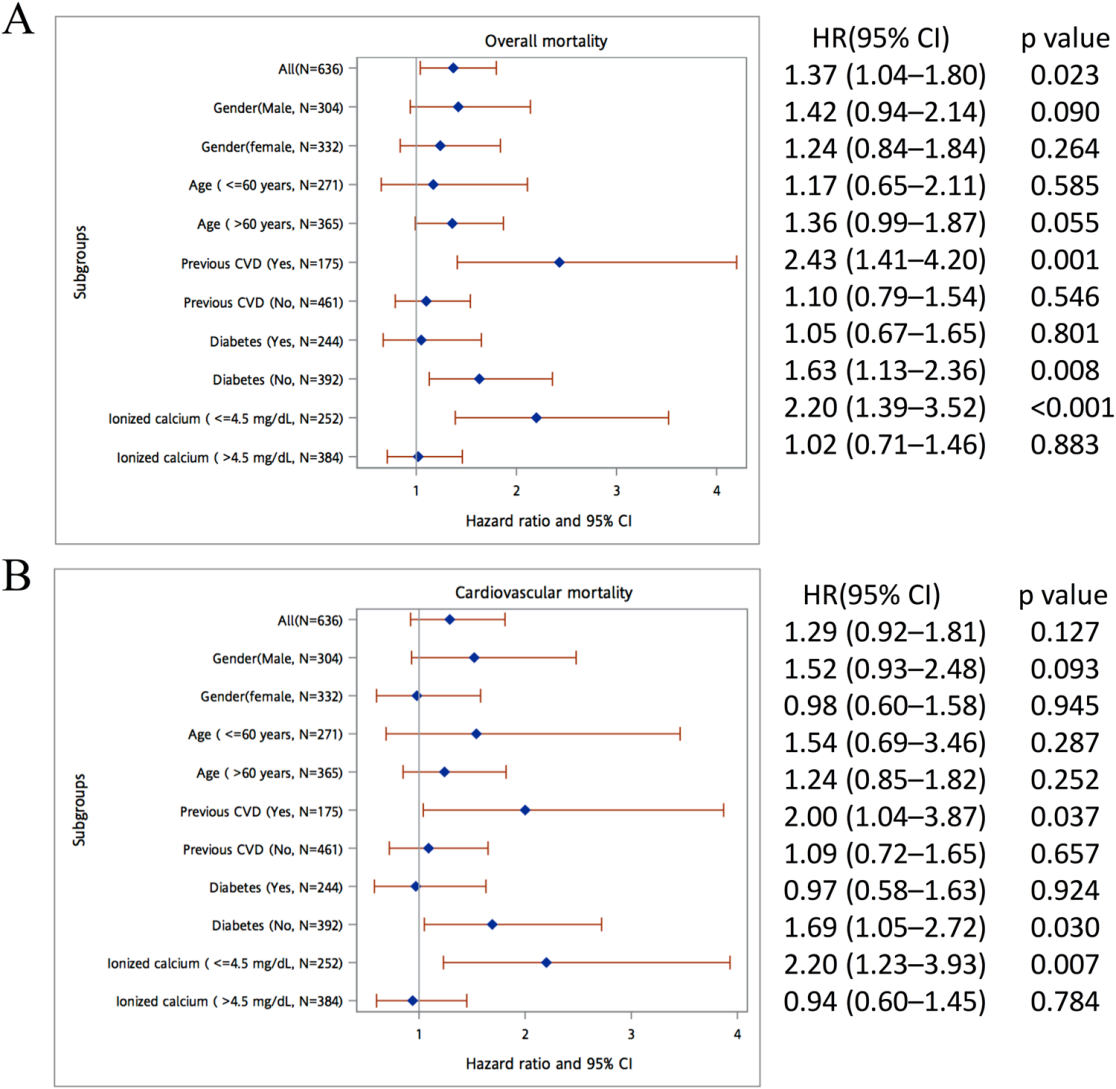
Subgroup analysis of the effect of serum aluminum levels (<6 and ≥6 ng/mL) on (**A**) all-cause mortality and (**B**) cardiovascular mortality among patients on chronic hemodialysis. The full model comprised adjusted variables including age; sex; hemodialysis vintage; diabetes mellitus; cardiovascular disease; cardiothoracic ratio; levels of creatinine, albumin, hemoglobin, ionized calcium, phosphate, and alkaline phosphatase; transferrin saturation; urea kinetics; as well as prescription of antiplatelet agents, renin–angiotensin blockers, beta-blockers, and lipid-lowering agents.

We also analyzed the association of the bivariate aluminum levels (<6 and ≥6 ng/mL) with all-cause and CV mortality stratified by covariates, including a history of DM or CVD, age (≤60 and >60 years), sex, and ionized calcium level (≤4.5 and >4.5 mg/dL). As shown in Fig. 3, after multivariable adjustment, the bivariate aluminum levels (<6 and ≥6 ng/mL) were significantly predictive of both all-cause and CV mortality in those with a CVD history, ionized calcium level of ≤4.5 ng/dL, and no history of DM.

## Discussion

In this study on 636 patients on CHD with the longest follow-up being 9 years, we found that a higher serum aluminum level (≥6 ng/mL) was independently associated with higher all-cause mortality after adjusting for potential confounders. Moreover, we found a linear association of ln(aluminum) level with all-cause death in patients on CHD. Interestingly, serum aluminum level was not independently associated with CV mortality and it might mediate the CV outcomes though the CTR level in patients on CHD. These findings expand our understanding of the association of serum aluminum levels with adverse outcomes in patients on CHD and emphasize that clinicians should be vigilant of potential early events even in patients in whom serum aluminum levels are within the acceptable range (below 20 ng/mL), as suggested by the KDOQI, and adopt early intervention approaches to prevent further aluminum accumulation in the body.

Aside from acute toxicity, aluminum accumulation will lead to gradual cellular damage. Aluminum has been reported to accumulate in all tissues in animals, preferentially in the liver, heart, bone, and brain^15^. Previous studies have provided several potential possible mechanisms to explain the association of serum aluminum levels with mortality in patients on CHD. One such mechanism is cellular oxidative stress induced by aluminum^16,17^, which may cause anemia and atherosclerosis in humans^18^, consequently increasing mortality in dialysis patients^19,20^. Another potential mechanism is chronic inflammation, as demonstrated by enhanced proinflammatory and proapoptotic gene expression in human brain cells exposed to aluminum sulfate^21^ and by the association of inflammatory markers with plasma aluminum levels in individuals with asthma^22^. Consequently, inflammation was found to be associated with an increased risk of mortality in patients on CHD^23,24^. Another mechanism that may underlie aluminum-associated mortality is protein–energy wasting induced by aluminum^25^. Hypoalbuminemia is recognized as a strong predictor of mortality in the CKD population^26^. The final consideration is the hazardous effect of aluminum in cardiac remodeling^7,27^, which the cardiotoxicity of aluminum might be attributable to oxidative stress and dysregulation of the intracellular redox system^18^. Some reports have found a significant association between heart damage and aluminum levels^28,29^. Undoubtedly, CVD is the leading cause of death in patients on CHD^30^. Taken together, these mechanisms might explain the significant association of serum aluminum levels with mortality of patients on CHD found in the current study. However, concise elucidation of the underlying mechanisms requires further investigations.

Adjustment for potential confounders by multiple linear regression analysis in the current study revealed that ln(aluminum) level was positively associated with HD duration, ionized calcium and alkaline phosphatase levels, and CTR. Patients with longer dialysis duration are expected to have higher aluminum levels owing to challenges in the efficient removal of aluminum by dialysis, leading to progressive accumulation over time. Aluminum will block calcium entry into the bone, consequently inducing osteomalacia^5^. Thereafter, hyperparathyroidism might be a mechanism to protect against aluminum-induced osteomalacia, perhaps by increasing bone turnover with elevated alkaline phosphatase levels^31^. Moreover, studies found evidence for aluminum-induced cardiac injury^28^ and cardiac complications^29^; higher aluminum loading in CHD patients was associated with cardiomegaly^7^ and cardiothoracic ratio (>0.5)^27^; cardiotoxicity of aluminum might be attributable to oxidative stress and dysregulation of the intracellular redox system^18^. Such clinical findings in previous studies can support our data.

An interesting finding from our data was that the higher serum aluminum level was not significantly associated with CV death in patients on CHD after adjusting the confounders but the association became significant after removing the parameter of CTR. Therefore, one hypothesis was made that higher serum aluminum level may contribute to the CV death through the higher CTR level, which a well-documented risk factor for poor prognosis in chronic dialysis^20,32–37^.

In subgroup analysis, aluminum levels had a significant predictive power for all-cause and CV mortality in patients with ionized calcium levels of ≤4.5 mg/dL, history of DM, and history of CVD. DM is a strong predictor of mortality in patients on CHD, which might negate the impact of aluminum levels on outcomes. A previous *in vitro* study indicated that aluminum inhibits the regeneration of reduced glutathione, thereby leading to oxidative damage^16^, which may induce atherosclerosis in humans^18^. Moreover, aluminum is harmful to cardiomyocytes. Such evidence supports our finding that patients with no history of CVD and those with lower calcium levels are more vulnerable to aluminum toxicity than those without.

The findings of the current study highlight the need for the close monitoring of aluminum levels in patients on CHD, even in those with aluminum levels below 20 ng/mL, as recommended by the KDOQI. Certain products containing aluminum that are commonly administered to dialysis patients include aluminum-containing phosphate binders and antacids, iron- and calcium-containing medications, calcitriol, vitamin B complex, erythropoietin, and insulin^10,11,38,39^. The first step in the prevention of aluminum toxicity is minimizing exposure to these medications, particularly aluminum-containing phosphate binders. Moreover, intensive hemodialysis (six times per week for 4–6 weeks) with a high-flux artificial kidney can efficiently remove aluminum^12,40^. Finally, the efficacy of early deferoxamine therapy still requires further evaluations to ascertain its benefits and risks. The KDOQI guidelines suggest the deferoxamine stimulation test and initiation of therapy in patients with serum aluminum levels of more than 20 ng/mL^12^.

Our study has several limitations. First, this was a single-center retrospective study; therefore, these findings may not be generalizable to all patient populations on CHD. Further multicenter studies as well as those including subjects from different ethnicities are needed to confirm our findings. Second, we only assessed baseline covariates to predict mortality, which might have resulted in biased estimates for associated variables that were time-varying predictors. Third, unstimulated serum aluminum level was used as the predictor, which may only reflect recent, limited exposure to aluminum and cannot provide information on the actual aluminum burden^41^. However, it is an easy and noninvasive approach to assess aluminum loading in patients on CHD, aside from bone biopsy and the deferoxamine stimulation test. Finally, data on the history of aluminum-containing medication exposure were unavailable in this cohort. However, we used serum aluminum level as the predictor, as higher frequency of aluminum exposure would lead to higher serum aluminum levels. Despite these limitations, the current study has several strengths. First, the follow-up period was long enough to include enough patients who reached the primary outcome. Second, adjustment was possible for several well-established factors related to mortality in dialysis.

In conclusion, we identified an independent association of a high aluminum level (≥6 ng/mL) and all-cause mortality in patients on CHD. Moreover, our results suggest a dose-dependent effect of serum aluminum levels on mortality and implicate that serum aluminum levels should be maintained as low as possible in patients on CHD. However, further study is still needed to clarify the benefits and harms of lowering serum aluminum levels in CHD patients in whom serum aluminum levels are within the acceptable range (below 20 ng/mL).

## Electronic supplementary material

Supplementary Dataset 1

## Data Availability

All data generated or analysed during this study are included in this published article (Supplementary Information files: S1 dataset).
